# Bacterial Communities in the Sediments of Dianchi Lake, a Partitioned Eutrophic Waterbody in China

**DOI:** 10.1371/journal.pone.0037796

**Published:** 2012-05-30

**Authors:** Yaohui Bai, Qing Shi, Donghui Wen, Zongxun Li, William A. Jefferson, Chuanping Feng, Xiaoyan Tang

**Affiliations:** 1 State Key Laboratory of Environmental Aquatic Chemistry, Research Center for Eco-Environmental Sciences, Chinese Academy of Sciences, Beijing, People's Republic of China; 2 College of Environmental Sciences and Engineering, The Key Laboratory of Water and Sediment Sciences (Ministry of Education), Peking University, Beijing, People's Republic of China; 3 School of Water Resource and Environmental Science, China University of Geosciences (Beijing), Beijing, People's Republic of China; 4 Kunming Institute of Environmental Science, Kunming, Yunnan Province, People's Republic of China; 5 Graduate School of Chinese Academy of Sciences, Beijing, China; Argonne National Laboratory, United States of America

## Abstract

Bacteria play an important role in the decomposition and cycling of a variety of compounds in freshwater aquatic environments, particularly nutrient-rich eutrophic lakes. A unique Chinese eutrophic lake - Dianchi - was selected for study because it has two separate and distinct basins, Caohai with higher organic carbon levels and Waihai with lower organic carbon levels. Sediment bacterial communities were studied in the two basins using samples collected in each season from June 2010 to March 2011. Barcoded pyrosequencing based on the 16 S rRNA gene found that certain common phyla, Proteobacteria, Bacteroidetes, Firmicutes and Chloroflexi, were dominant in the sediments from both basins. However, from the class to genus level, the dominant bacterial groups found in the sediments were distinct between the two basins. Correlation analysis revealed that, among the environmental parameters examined, total organic carbon (TOC) accounted for the greatest proportion of variability in bacterial community. Interestingly, study results suggest that increasing allochthonous organic carbon could enhance bacterial diversity and biomass in the sediment. In addition, analysis of function genes (*amoA* and *nosZ*) demonstrated that ammonia-oxidizing bacteria (AOB) were dominant in sediments, with 99% belonging to *Nitrosomonas*. Denitrifying bacteria were comparatively diverse and were associated with some cultivatable bacteria.

## Introduction

Although eutrophication is a natural process that may become more pronounced as aquatic ecosystems age, human activities can greatly accelerate this process [1]. In China, eutrophication has become particularly problematic in many freshwater lakes as a consequence of rapid economic development. Dianchi is a representative eutrophic lake as it is located in a densely populated city (Kunming) and is subject to substantial organic carbon and nitrogen loading. An increase in the discharge of untreated domestic and industrial wastewater directly into Dianchi Lake and its tributaries has caused a significant rise in the number and extent of Cyanobacterial blooms. Dianchi Lake is separated into two basins, each with a distinct level of organic carbon pollution. This limnological dichotomy of Dianchi Lake allows for the exploration of variations of the unique micro-ecosystems, along with inputs of allochthonous organic carbon.

Prokaryotes, especially bacteria, play a dominant role in nutrient recycling and decomposition of chemical compounds in eutrophic lakes [2], [3]. Therefore, a shift in bacterial communities may be one of the most sensitive indicators of environmental change in eutrophic lakes [4]. Previously, bacterial communities were monitored using fingerprinting or clone library techniques, which have documented limitations. In recent years, 454 Pyrosequencing, based on 16 S rRNA genes or function genes, has been widely used to obtain more comprehensive information about microbial diversity. This technique eliminates the laborious step of conducting clone library and can simultaneously sequence a large numbers of individual samples [5], [6]. Results obtained from pyrosequencing methods have altered traditional views of microbial diversity and composition in the ocean [7], drinking water [8], soil [9] and the human gastrointestinal tract [10], etc. But few studies used this method to elucidate the bacterial community in eutrophic lakes [11], [12], [13], [14], [15].

Lake sediment deposits can function as either a source or a sink for a large number of essential nutrients involved in the eutrophication process [16]. Therefore, investigations of sediment bacterial communities can lead to a better understanding of aquatic ecosystems [17]. The goal of the present study was to compare the sediment bacterial communities of Caohai Lake (high organic carbon level) and Waihai Lake (low organic carbon level), using both barcoded pyrosequencing and traditional molecular methods (e.g., Terminal restriction fragment length polymorphism (T-RFLP) and clone library). The community parameters examined were total bacteria (16 S rRNA), nitrifiers (*amoA*) and denitrifiers (*nosZ*). The results of this research provide new insights into, and valuable references for, the bacterial communities in eutrophic lakes. Moreover, the information generated will help guide government regulatory action to reduce pollutant loading to lakes and other aquatic ecosystems.

## Materials and Methods

### Ethics Statement

No specific permits were required for the described field studies. The location is not privately-owned or protected in any way and the field studies did not involve endangered or protected species.

### Site description and sediments collection

Dianchi Lake is located in southwestern China in the capital of Yunnan Province. It covers about 308.6 km^2^. The water depth is approximately 5.03 m, with a maximum water depth of 11.35 m. The hydrological retention time is 2–3 years. Lake water temperature ranges from 9.8 to 24.5°C, with an annual average of about 16.0°C. In autumn and winter wind strength and frequency increases, ensuring that the lake does not stratify [18]. Twenty-nine streams flow into Dianchi Lake. Caohai (3% of total area) receives the greatest contaminant load from industry and agriculture, resulting in a more serious pollution problem than Waihai [19]. Based on 2009 and 2010 monitoring data [20], [21], pH in both Caohai and Waihai ranged from 6 to 10 and dissolved oxygen (DO) was stable at between 6 and 7 mg/l. Biochemical oxygen demand (BOD, 7.9–17.0 mg/l), ammonia nitrogen (NH_3_-N, 4.3–16.1 mg/l), total phosphorus (TP, 0.3–2.2 mg/l) and total nitrogen (TN, 6.2–19.4 mg/l) in Caohai were all higher than what has been measured in Waihai (BOD, 2.3–5.4 mg/l; NH_3_-N, 0.2–0.4 mg/l; TP, 0.1–0.2 mg/l; TN, 1.7–3.4 mg/l).

Sediment samples were collected from two sites in Dianchi Lake, approximately mid-lake in each of the separate basins (Fig. S1). Water depths at the sampling locations were 3 m in Caohai and 8 m in Waihai. Each location was sampled four times, all within an area of 25 m^2^, in June, September and November 2010 and March 2011. The surface sediment samples were collected with a stainless steel grab sampler, placed in clean containers and stored with ice during transport to the laboratory, where they were then stored in a freezer at −80°C.

### Physical and Chemical analysis

Sediment surface temperatures were measured with a thermometer during each sampling trip. Sediment samples were dried with a freeze drier (Christ Alpha 1–2 LD plus, German) and the water content of each sample was calculated. For NH_3_-N, nitrate nitrogen (NO_3_^−^-N), and nitrite nitrogen (NO_2_^−^-N) analysis, 1–2 g dried samples were extracted with 40 ml 2 M KCl for 2 h in a shaker. The extract was then filtered through a 0.45 µm membrane filter prior to analysis. The concentrations of NH_3_-N, NO_2_^−^-N, and NO_3_^−^N were measured using standard methods [22] with a UV-VIS spectrometer (Shimadzu UV2401, Japan). Sediment pH was determined using a sediment to water ratio of 1∶2.5 (w/w). TOC (Total organic carbon) was measured with a titration method [23].

### DNA extraction

DNA was extracted from 0.5 g fresh sediment using the Power soil DNA isolation kit (Mobio, USA) according to the recommendation of manual. Three replicate DNA extractions of individual sediment cores were pooled prior to PCR amplification. All total DNA samples were stored at −80°C in a freezer for following molecular application.

### Barcoded pyrosequencing for 16 S rRNA

The V1–V3 region of the 16 S rRNA gene was amplified from sediment DNA by PCR using barcoded universal primers 8F and 533R containing the A and B sequencing adaptors (454 Life Sciences). The fusion PrimerA-8F was 5′- cgtatcgcctccctcgcgccatcagAGAGTTTGATCCTGGCTCAG -3′ where the sequence of the A adaptor is shown in lowercase letters. The reverse fusion Primer B-533R was 5′- ctatgcgccttgccagcccgctcagTTACCGCGGCTGCTGGCAC -3′ where the sequence of the B adaptor is shown in lowercase letters. The ten base pair barcode unique to each sample embedded in the Primer B-533R set.

The PCR amplification was performed in a ABI9700 thermocycler (ABI, Foster City, USA) using the program 95°C for 2 min; 25 cycles of 95°C for 30 s, 55°C for 30 s, 72°C for 30 s; 72°C for 5 min; finally kept at 10°C. Triplicate positive PCR products were pooled and purified with AxyPrep DNA Gel Extraction Kit (Axygen, USA). The DNA concentration of the purified amplicons was measured by TBS-380 Fluorometer (Turner Biosystems, CA, USA). Prior to sequencing, the amplicons from each reaction mixture were mixed in equal amounts based on concentration and subjected to emulsion PCR, and amplicon libraries were generated as recommended by 454 Life Sciences. Sequencing was performed from the primer B end using the 454/Roche B sequencing primer kit using a Roche Genome Sequencer GS-FLX according to the protocol.

Pyrosequencing flowgrams were converted to sequence reads using Mothur software [24] and were then analyzed using QIIME standard pipeline [25]. Sequence reads were initially filtered and denoised for removing low quality or ambiguous reads. Then the treated sequences were checked with ChimeraSlayer [26] and the putative chimeric sequences were excluded for further analysis. The remaining set of high quality 16 S rRNA sequences were clustered into OTUs using UCLUST [27], with 97% sequence identity threshold. OTUs-based analysis was performed using QIIME pipeline. Briefly, representative sequences from each OTU were aligned using PyNAST [28] and the Greengenes [29] database. Taxonomy was assigned using the Ribosomal Database Project (RDP) classifier (minimum confidence of 80%) [30]. Rarefaction and Alpha diversity statistics including library coverage [31], nonparametric richness Chao 1 [32], and Shannon index, were calculated for each sample. The weighted and unweighted UniFrac distance metrics [33] were used to compare community diversity. Unweighted Pair Group Method with Arithmetic mean (UPGMA) using average linkage was used to interpret the distance matrix of each sample.

All original and non-chimaeric 454 sequences are archived at NCBI Sequence Read Archive (SRA) under accession SRP009448.

### T-RFLP for *amoA* and *nosZ*

PCR amplification of the *amoA* and *nosZ* gene from the total DNA was performed using the 6-FAM fluorescent tagged primers at 5′ end of the forward primer (Table S1). The target PCR products were purified using the Qiaquick PCR purification kit (QIAGEN, Germany). Restriction digests of the purified PCR products were produced using Sau 96 Ι (for *amoA*) and *Hha*Ι (for *nosZ*) enzymes (MBI, Fermentas, USA) according to the recommendation. The digested products were desalted by ethanol precipitation [34] and analyzed using an ABI PRISM 3700 DNA analyzer (Applied Biosystems, Foster City, CA) at Genescan mode. The GS500 Liz internal size standards (Applied Biosystems) were employed. T-RFLP electropherograms were inspected using the Peak Scanner software (Applied Biosystems). Terminal restriction fragment sizes >35 and with peak heights >50 in relative fluorescence units were marked for subsequent statistical analysis. T-RFLP profiles were pre-treated using the *T-REX* online software [35] and the treated data were then imported into the program PAST (Ver. 2.12, http://folk.uio.no/ohammer/past/) for hierarchical cluster analysis.

### Cloning libraries for *amoA* and *nosZ*

PCR amplifications for the *amoA* and *nosZ* genes were performed with the widely used prime pairs (Table S1).

PCR products were purified with the Qiaquick gel extraction kit and cloned into pGEM-T Easy vectors (Promega, USA). The recombinant plasmids were transformed into competent *E. coli* JM109. Fifty positive clones for both genes were randomly selected and sequenced using an ABI 3730*xl* DNA Analyzer (Applied Biosystems, USA). Low -quality ends and vector contaminants were removed by the software SeqMan Pro software (DNASTAR). Ambiguous sequences were checked manually and excluded from further analysis. ∫-LIBSHUFF was used to compare the gene sequences of two libraries and determine if they were significantly different [36]. Operational taxonomic units (OTUs) were defined as groups where the sequence similarities were greater than 98%. Rarefaction and diversity statistics including library coverage, nonparametric richness Chao 1, and Shannon index, were calculated using the Mothur software [24]. The OTU sequences combined from Caohai and Waihai were translated to amino acid sequences with Bioedit software and blasted against published gene sequences in the National Center for Biotechnology Information (NCBI) database. Phylogenetic trees were constructed using the neighbor-joining method with the software MEGA 5 [37].

All OTU sequences of *amoA* and *nosZ* are deposited in the NCBI database under the accession numbers JQ081008 to JQ081020 and JQ081021 to JQ081062, respectively.

### Real-time PCR assay

Bacterial 16 S rRNA, *amoA*, and *nosZ* genes were quantified by an ABI 7500 fast real-time PCR system based on SYBR Green I method. The primer pairs and the thermal programs of the PCR amplification were described in the Table S1. Plasmid standards containing the target genes were generated from the appropriate positive clones obtained from clone libraries. The plasmid DNA concentration was determined by an Eppendorf BioPhotometer Plus (Gemany). The copy number of each target gene was calculated directly from the concentration of the extracted plasmid DNA. Ten-fold serial dilutions of the standard plasmid DNA were subjected to a real-time PCR assay in triplicate to generate an external standard curve and to check the amplification efficiency. The specificity of the PCR for each target gene was checked using melting curve analysis and gel electrophoresis.

The qPCR assay efficiencies were 99.5% for16 S rRNA, 92% for *amoA*, and 95% for *nosZ*. The correlation coefficients (*R* ^2^) were all over 0.999.

### Statistical analysis

To correlate bacterial community and composition with sediment properties, Spearman's rank were determined by using the SPSS 18.0 (SPSS Inc., Chicago, IL, USA). In order to reveal the relationship between the community composition (dominant phyla and proteobacterial classes) and sediment properties, their data matrixes were tested under a detrended correspondence analysis (DCA) model using CANOCO 4.5.1 (Biometris-Plant Research International, Wageningen, The Netherlands). Results showed the length of the first gradient was found to be less than 3 SD, and hence linear models were constructed. Hypothesis testing was performed using Principal components analysis (PCA) and Redundancy analysis (RDA) with Monte Carlo tests (CANOCO 4.5.1).

## Results

### Sediment properties

Sediment properties of samples from each sampling site are shown in Table S2. Seasonal sediment temperatures varied from 10.5 to 22.9°C. Sediment pH ranged from 6.2 to 6.4. Water content in Caohai samples (82.3–91.5%) was higher than in Waihai samples (63.8–86.7%). The sediment TOC concentrations for Caohai (515–10,915 mg/g dry weight) and Waihai (350–965 mg/g dry weight) demonstrated that organic carbon loading to Caohai is more serious than to Waihai. Both Caohai and Waihai sediments revealed high concentrations of nitrogenous compounds, especially nitrate (up to 1732 mg/g in Caohai and 2795 mg/g in Waihai). Freeze-dried sediments from Caohai were black while those from Waihai were yellow (Fig. S2). In general, the concentrations of sediment pollutants in the autumn and winter samples were always higher than those measured in spring and summer samples.

### Pyosequencing for 16 S rRNA

The pyrosequencing analysis of 16 S rRNA gene amplicons from eight sediment samples produced 122,263 reads, leaving 85,975 reads after quality filtering and removal of chimeric sequences. The average sequence length was of 449 nucleotides, excluding the adaptor and barcode primer sequences. The sequences could be assigned to 9596 operational taxonomic units (OTUs) at a 97% sequence identity threshold. Species richness, coverage and diversity estimations were calculated for each data set (Table 1). Good's coverage revealed that these libraries represented the majority of bacterial 16 S rRNA sequences present in each sediment sample, with values ranging from 83.6% to 95.6%. Chao 1 values and rarefaction curves (Fig. S3) clearly showed that the bacterial richness in Caohai sediments was significantly higher than in Waihai sediments. Among different seasons, the highest richness values in Caohai and Waihai occurred in March and June, respectively. The richness values in September were lowest in both Caohai and Waihai. The Shannon index, a metric for community diversity, revealed a comparatively higher level of overall biodiversity in the sediments of Caohai. As with richness, highest diversities were found in March (Caohai) and June (Waihai).

**Table 1:** Library coverage estimations and sequence diversity of 16 S rRNA.

Sample	No. of raw sequences	No. of Filtered sequences	No. of OTU (>97% identity)	% Coverage	Chao 1 value	Shannon index
Caohai (Mar.)	13819	9419	2588	85.4%	3992(3700, 4335)	9.85
Caohai (Jun.)	9216	6033	1775	83.6%	3473(3197, 3802)	9.53
Caohai (Sept.)	17453	13156	2118	92.8%	2616(2409, 2868)	8.95
Caohai (Dec.)	13679	8497	1832	88.5%	2889(2655, 3172)	8.56
Waihai (Mar.)	17756	13071	1186	95.4%	1530(1359, 1753)	6.09
Waihai (Jun.)	16045	11716	2330	90.6%	3045(2815, 3321)	9.13
Waihai (Sep.)	15993	11671	1111	95.6%	1431(1284, 1624)	6.11
Waihai (Dec.)	18302	12412	1967	92.1%	2536(2317, 2805)	8.08

Library coverage was calculated as C = 1-*n*/*N*, where *n* is the number of OTUs without a replicate, and *N* is the total number of sequences. The numbers in parentheses are lower and upper 95% confidence intervals for the Chao 1 estimators. The Shannon index = 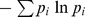, where *p_i_* = *n_i_*/*N*, *n_i_* is the number of OTUs with *i* individuals, and *N* is the total number of individuals.

**Figure 1:**
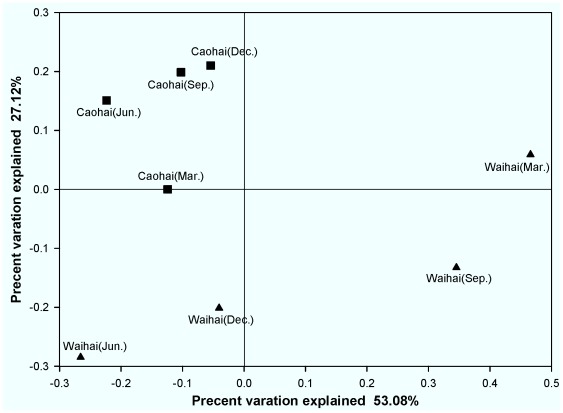
Community analysis using principal coordinate analysis (PCoA) of weighted UniFrac distance matrix.

We compared bacterial community composition in sediments using the weighted UniFrac distance metric (Fig. 1) and unweighted UniFrac distance metric (data not shown). These two analyses showed strong clustering of samples from Caohai, and a dispersed distribution of Waihai samples, probably indicating that the sediment bacterial communities in Waihai were more susceptible than in Caohai under different seasons. From Hierarchical cluster analysis (Fig. S4), we concluded that sampling location was the primary factor accounting for variability in bacterial community structure, as Caohai or Waihai sediment samples were clustered into their respective groups.

**Figure 2:**
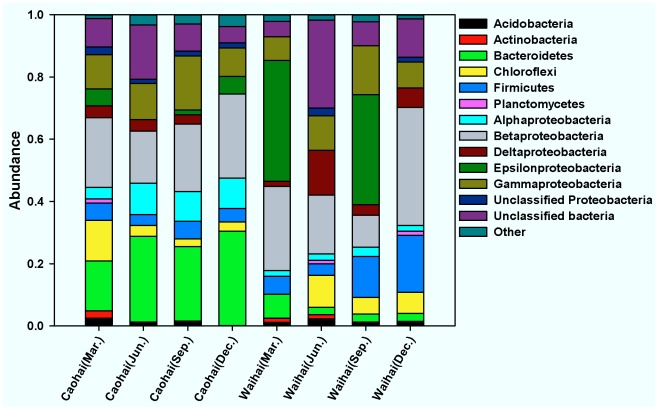
Relative abundances of dominant phylogenetic groups in sediments derived from Caohai and Waihai sampling sites. Phylogenetic groups accounting for <1% of all classified sequences are summarized as “other” in the figure.

Of our filtered sequences, we found 27 different bacterial phyla across all sediment samples (Caohai, 25; Waihai, 24) with the RDP classifier. The dominant groups (greater than 1% abundance) of each sample are displayed in Fig. 2. Proteobacteria (44%–77%) was the most abundant phylum across all samples, and was relatively higher in Waihai samples. The proteobacteria in the two sites consisted of five classes (Alphaproteobacteria, Betaproteobacteria, Gammaproteobacteria, Epsilonproteobacteria and Deltaproteobacteria) plus unclassfied proteobacteria. Betaproteobacteria was dominant in most of the samples. Bacteroidetes was another abundant phylum in Caohai sediments (16–31%), but was lower in Waihai sediments (2.3–7.7%). Firmicutes and Chloroflexi were also abundant and present in all Caohai and Waihai sediment samples. Seasonal changes in the bacterial community were apparent, as shown by the sharp decline in the abundance of Epsilonproteobacteria. In Waihai sediments, this taxon changed from 38.8% (March) and 35.3% (September) of the community to 0.2% (December) and 0.02% (June). Unclassified bacteria significantly increased in the summer sediments of Caohai and Waihai.

A total of 415 bacterial genera were identified in our dataset. The numbers of genera in each sample were 231, 198, 269 and 207 in Caohai sediments, and 219, 142, 153 and 141 in Waihai sediments for March, June, September and December, respectively. In each season, taxa richness was greater in Caohai sediments. The dominant genera (top 10 of each sample) are shown in the Fig. S5. Many of the unclassified genera belonged to certain families, orders, or classes were present in the sediment samples, also reflecting the taxonomically complex environment of Dianchi sediments. There were notable differences in genera between Caohai and Waihai sediments. The most common bacterial genera in Caohai sediments belonged to Bacteroidetes or Chloroflexi, while proteobacteria had the greatest abundance in Waihai sediments. The most abundant classified genera of bacteria in Caohai sediments were *Dechloromonas* (Mar., 7.8%) and *Flavobacterium* (Jun., 11.5%; Sep., 7.4%; Dec., 19.7%), but were *Sulfuricurvum* (Mar., 9.4%), *Thiobacillus* (Jun., 8.1%), *Bacillus* (Sep., 10.4%), and *Massilia* (Dec., 17.6%) in Waihai sediments.

### Diversity of *amoA* and *nosZ*

Ammonia-oxidizing bacteria (AOB) and denitrifying bacteria were investigated by utilizing *amoA* and *nosZ* gene fragments, respectively, to track their abundance and composition. T-RFLP, based on the fluorescent tag for DNA fragments, was used in this experiment to track community variation. The T-RFLP profiles for *amoA* and *nosZ* showed that most of the dominant peaks of Caohai and Waihai sediment samples were identical in length under different seasons, but varied in peak area or height (data not shown). Cluster analysis (Fig. S6) showed the T-RFLP profiles of sediment samples grouped into un-ordered clusters. These results indicated that the AOB and denitrifying bacteria communities might change significantly in quantity but not in species composition from season to season. In addition, we found that PCR amplification for *amoA* and *nosZ* gene fragments obtained from some sediment DNA had a weak gel band, probably owing to the low gene copy numbers of *amoA* and *nosZ*, or to DNA inhibitors in the samples. Taking PCR performance and T-RFLP results into account, only December samples from Caohai and Waihai were selected for the construction of the clone library to explore community composition.

**Figure 3:**
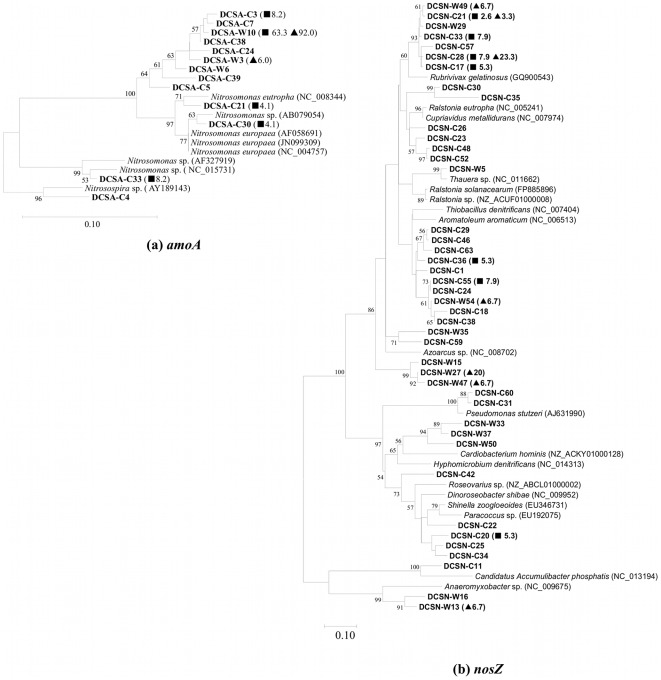
Phylogenetic trees with the (a) *amoA* and (b) *nosZ* representative sequences (OTUs) from Dianchi Lake sediments (Sampled in Dec. of 2010). The numbers on the branch nodes represent percentage of bootstrap resamplings based on 1000 replicates (only ≥50% are shown). The scale bar indicates the number of nucleotide substitutions per site. The relative abundance of each OTU (comprising 2 or more clones) in the *amoA* or *nosZ* clone library is shown in parentheses. Additional symbols for abundance are (▪) Caohai and (▴) Waihai.

From *amoA* libraries we obtained 49 filtered sequences from Caohai sediments and 50 sequences from Waihai sediments. Pairwise comparison of the two libraries using ∫-LIBSHUFF revealed that the sequences were not significantly different (*p* = 0.7118). The number of OTUs (>98% identity), estimated species coverage, richness and diversity were calculated for the two libraries (Table S3). The Shannon indices of the Caohai (1.53) and Waihai (0.32) libraries and the rarefaction curve (Fig. S7) suggest the *amoA* diversity in Caohai sediment was significantly higher than in Waihai sediment. The predicted bacterial species present in the two libraries are 19 (Caohai) and 3 (Waihai). Thirteen unique OTUs were identified from two library sequences, as shown in the combined phylogenetic tree (Fig. 3(a)). The homology between these OTUs and sequences from the NCBI database ranged from 96% to 100% at the amino acid level. All Caohai *amoA* sequences and most Waihai *amoA* sequences (98%) were grouped in the *Nitrosomonas* genus. The DCSA-W10 sequence appeared to be related to *nosZ* of *N. europaea* (91%, amino acid level) and was present in significant proportions in both Caohai sediment (63.3%) and Waihai sediment (92.0%) samples.

For *nosZ* libraries, only 76.0% of the sequences in Caohai and 60.0% of the sequences in Waihai remained after quality filtration (Table 3). In contrast to *amoA*, the two *nosZ* libraries were significantly different (*p* = 0.0003) according to ∫-LIBSHUFF analysis. Shannon indices in Caohai (3.21) and Waihai (2.45) libraries, and the rarefaction curves (Fig. S7), also demonstrated the higher diversity of *nosZ* in Caohai sediment. The predicted bacterial species present in *nosZ* libraries were 85 (Caohai) and 24 (Waihai), which were higher than the *amoA* libraries. The combined phylogenetic tree based on the *nosZ* amino acid sequences (Fig. 3(b)) showed the distinct distribution of OTUs between the two libraries. These OTUs shared 75–97% similarities with known sequences registered in the NCBI database. Two OTUs found in two libraries, DSCN-C28 and DSCN-C21, were identical to *nosZ* of *Rubrivivax gelatinosus*, with 89% and 87% similarity, respectively.

### Quantitative analysis of 16 S rRNA, *amoA*, and *nosZ*

**Figure 4:**
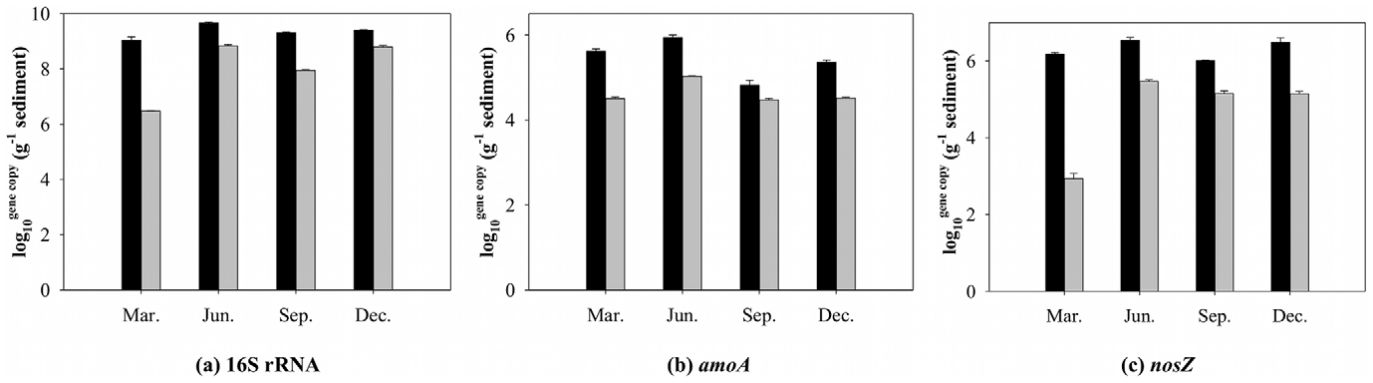
Quantitive analysis of 16 S rRNA, *amoA*, and *nosZ* gene in the sediments (computed in dry weight). Black bars represent Caohai and grey bars represent Waihai. Error bars represent standard deviation from three independent experiments.

The qPCR results showed that the abundances of bacterial 16 S rRNA, *amoA* and *nosZ* genes in Caohai sediments were all higher than those in Waihai sediments (Fig. 4). The *amoA*/16 S rRNA ratio in Caohai and Waihai sediments ranged from 10^−3^ to 10^−4^, and *nosZ*/16 S rRNA ranged from 10^−2^ to 10^−5^. The quantity variations of 16 S rRNA, *amoA* and *nosZ* in four seasons were similar in Caohai and Waihai sediments, with the highest values found in the June samples. The gene copies of the December samples in both Caohai and Waihai were not the lowest, indicating there were other factors, aside from temperature, that affected the quantity of microbes.

### Relationship between bacterial community and sediment properties

Correlation results (Table S4) showed that the copy number of 16 S rRNA had a significant positive correlation with TOC (R = 0.738; p<0.05). Other metrics, including bacterial richness (Chao 1), diversity (Shannon index), and the abundances of *amoA* and *nosZ*, were not significantly correlated with sediment properties. At the phylum and proteobacterial class level (Table S5), the relative abundances of Actinobacteria (R = −0.791), Bacteroidetes (0.714) and Alphaproteobacteria (0.762) in the sediments were significantly correlated with TOC, while Betaproteobacteria (−0.833) and Gammaproteobacteria (0.738) were correlated with temperature (p<0.05 in all cases). Eplisonproteobacteria was not found to be significantly correlated with any factors.

**Figure 5:**
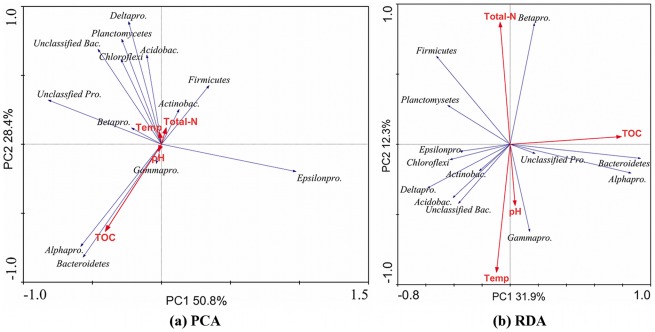
Principal components analysis (PCA) and Redundancy analysis (RDA) with of bacterial communities as affected by sediment properties, based on the relative abundance of dominant bacterial phyla and proteobacterial classes. Total-N represents NH_3_-N+NO_2_^−^-N+NO_3_^−^-N. Abbreviations in figure: Temp, temperature; *Actinobac.*, *Actinobacteria*; *Alphapro.*, *Alphaproteobacteria*; *Betapro.*, *Betaproteobacteria*; *Deltapro.*, *Deltaproteobacteria*; *Gammapro*.,*Gammaproteobacteria*; *Acidobac.*, *Acidobacteria*; Unclassified bac., Unclassified bacteria; Unclassified *Pro.*, Unclassified *Proteobacteri*a. *Epsilonpro*., *Epsilonproteobacteri*a.

PCA and RDA results demonstrated that, of the parameters examined, TOC accounted for the greatest amount of variability in the bacterial community (Fig. 5). PCA and RDA analyses established a similar correlation between bacterial community parameters and sediment properties (compare to Table S5).

## Discussion

The physical, chemical and biological characteristics of pelagic sediment are considerably more suitable than those of littoral sites to reflect the overall environmental quality of Dianchi Lake. We therefore chose two typical pelagic sites as target locations to elucidate variations in the sediment bacterial communities, as well as relationships of the biotic metrics to sediment properties. Previous studies on Dianchi Lake focused primarily on pollutant properties [38], [39], algae [40], [41], or fungi [42], although one investigation did explore the bacterial community in the polluted water near the shore of Dianchi Lake [43]. Among such studies of other eutrophic lakes, few reports used pyrosequencing to analyze the sediment microbial community.

### Bacterial diversity and composition in Dianchi Lake

In our study, bacterial phylogenetic assignments of pyrosequencing results (27 phyla and 415 genera) revealed a high level of bacterial diversity, with sediments usually harboring the most diverse bacterial populations in the lake [4], [44]. Compared with previous studies based on clone library [4], [45] or fingerprinting method [3], [46], we identified a much more diverse bacterial population. These data indicate that pyrosequencing is a powerful and effective tool for microbial community analysis. Proteobacteria, Bacteroides, Firmicutes and Chloroflexi were dominant in Dianchi Lake sediments (Fig. 2), and are common phyla in eutrophic lakes [17], [47]. Cyanobacteria that are dominant in the water column, particularly near shore [43] were not abundant (up to only 0.13%) in the sediments. This low number may be due to their light requirements, as suggested in a previous study [48]. Usually Betaproteobacteria occur almost exclusively in freshwater environments [49]. Interestingly, our pyrosequencing results showed that Epsilonproteobacteria, which has been implicated in chemoautotrophic production [50], was dominant in two of the Waihai sediment samples. While Epsilonproteobacteria has been observed as a dominant taxon in oil field [51], it has seldom been identified as dominant in eutrophic lake sediments. Further assignment revealed that the Order Hellcobacteraceae, from the Campylobacteraceae lineage, was abundant in two Waihai sediment samples (Fig. S5). It has not been previously documented in similar studies.

To investigate which type of microbe dominated ammonia oxidation, we first performed PCR with three universal primer pairs to amplify the ammonia oxidizing genes corresponding to ammonia-oxidizing bacteria (AOB) [52], ammonia-oxidizing archaea (AOA) [53], and anaerobic ammonium oxidation bacteria (anammox) [54]. The results showed that no obvious PCR amplification was effective in exhibiting the ammonia-oxidizing genes of AOA and anammox in Caohai and Waihai sediments. Therefore, the *amoA*-representing the AOB community may take the premier role in the nitrification in sediments, since it could be amplified using PCR. The AOB in Dianchi sediments have comparatively lower diversity compared to Donghu [55] and Taihu [56], two eutrophic lakes located in China. *Nitrosomonas-*related AOB forms a major component of the ammonia-oxidizing communities in the Dianchi sediments. This genus was often reported to be abundant in freshwater environments, particularly in activated sludge, biofilm reactors and biofilters [57]. When conducting a combined phylogenetic tree with OTU sequences from both sediment and water (Fig. S8), we found some close resemblance, suggesting the similarity of AOB community structure in water and sediment.

Denitrifying bacteria in Dianchi sediments, which were affiliated with the *α*, *β*, and *γ* subclass of the class Proteobacteria, have greater diversity than those in Dianchi lakeshore water [43]. The cloning library also suggests that many denitrifying bacteria in Dianchi Lake are related to some cultured denitrifiers such as the *Pseudomonas*, *Paracoccus* and the *Thauera* group (Fig. 3b). These results are somewhat inconsistent with another study in ocean sediment where the *nosZ* genes clustered monophyletically and were not associated with cultivable denitrifying bacteria [58], indicating salinity may be a key factor controlling the composition of the denitrifying bacterial community [59].

### Relating community structure to sediment properties

The quantity (Fig. 4), richness and diversity (Table 1 and Table S3) of total bacteria, AOB and denitrifying bacteria in Caohai sediment were all higher than those in Waihai. The difference suggests that a higher organic carbon level might lead to higher bacterial biomass and diversity in the sediment.

Previous studies demonstrated that pH, temperature and certain inorganic and organic contaminants are the most important drivers of the bacterial communities in sediment or soil [57], [60], [61]. Among pH, certain compounds (TOC, NH_3_-N, NO_2_^−^-N, and NO_3_^−^-N) and temperature in our study, TOC was found to be the primary driver that affected variability of bacterial density (qPCR for 16 S rRNA) and composition (phyla) in Dianchi Lake sediments. Of the other measured metrics, (i) pH was seasonally quite stable and seasonal temperature change was relatively small because of the location of Dianchi Lake (latitude of around 24°–25°N), (ii) pollutants discharged from various industries and agriculture, as well as precipitation (which can affect point and nonpoint source in-flow) differed from season to season (thus potentially affecting TOC concentration), and (iii) the ratios of C/N (roughly reflected in TOC/(NH_3_-N+NO_2_^−^-N+NO_3_^−^-N)) in Caohai (0.7–7.6) and Waihai (0.3–0.7) were both much lower than 20∶1 to 30∶1, which is regarded as the optimum range for bacterial growth. Therefore, the carbon source (reflected in TOC) was the limiting factor for bacterial growth. Our data strongly indicate that the shift of TOC concentrations was the primary factor responsible for changes in the pelagic sediment bacterial community in Dianchi Lake. This result is different from what was found in another Chinese eutrophic lake (Taihu), which showed that temperature and total phosphate were the dominant factors affecting the sediment bacterial community [62].

Gudasz et al. [63] reported in a recent study that increasing allochthonous organic carbon in the water column did not enhance sediment bacterial metabolism. But the study did not discuss the relationship between allochthonous organic carbon in sediment and sediment bacterial metabolism. The sources of TOC in Dianchi Lake are mainly industrial and domestic wastewater, stormwater runoff and agricultural point and nonpoint source inputs. Using data from both Caohai and Waihai, we found that allochthonous organic carbon in sediment was positively correlated with sediment bacterial biomass. Gudasz et al. [63] reported that bacterial biomass was positively correlated with bacterial production and organic matter mineralization. Therefore, it is reasonable to conclude that increasing allochthonous organic carbon in lake sediment could enhance sediment bacterial metabolism. This is consistent with several previous studies of lake water column communities, which have shown that raising the inputs of terrestrial organic carbon also increases bacterial metabolism [64], [65].

## Supporting Information

Figure S1 **Map of sampling sites from Dianchi Lake, Yunnan Province, China.** The red line represents the dam that separates the Caohai (North part) and Waihai (South part). Sampling site of Caohai (▴):24°58′48.55″N, 102°38′31.92″E; Sampling site of Waihai (▾): 24°49′48.00″N, 102°42′47.00″E. The map was obtained from Google map and edited by the software Mapinfo 7.0.
(PDF)

Figure S2 **Photo of freeze-dried sediments (Sampling in Dec. 2010).**
(PDF)

Figure S3 **Rarefaction curve of pyrosequencing libraries.**
(PDF)

Figure S4 **Hierarchical cluster analysis for barcoded pyrosequencing data based on Unweighted Pair Group Method using average linkage.** The figure was plotted with FigTree software.
(PDF)

Figure S5 **Relative abundances of the most abundant genera (top 10 of each sample) in Caohai and Waihai sediments.** The heatmap was plotted with R program (http://www.r-project.org).
(PDF)

Figure S6 **Dendrogram for hierarchical cluster analysis based on group-average linking of Bray-Curtis similarities calculated from the binary (0 or 1) data of the (a)** ***amoA*** **and (b)** ***nosZ*** **gene T-RFLP profiles.** The symbol CH represents Caohai and WH represents Waihai.
(PDF)

Figure S7 **Rarefaction curve of** ***amoA*** **and** ***nosZ*** **clone libraries.**
(PDF)

Figure S8 **Phylogenetic trees with the** ***amoA*** **representative sequences (OTUs) from Dianchi Lake sediments (bold letter) and lakeshore water** [**6**] **.** The numbers on the branch nodes represent percentage of bootstrap resamplings based on 1000 replicates (only ≥50% are shown). The scale bar indicates the number of nucleotide substitutions per site.
(PDF)

Table S1 **PCR primer pairs and thermal programs used in this study.**
(PDF)

Table S2 **Physico-chemical characteristics of the sediments of Dianchi Lake.**
(PDF)

Table S3 **Library coverage estimations and sequence diversity of** ***amoA*** **and** ***nosZ*** **.**
(PDF)

Table S4 **Correlations between richness (Chao 1 values), diversity (Shannon index), abundance of 16 S rRNA,** ***amoA*** **,** ***nosZ*** **(qPCR) and sediment properties.**
(PDF)

Table S5 **Correlations between the relative abundances of the most abundant bacterial phyla and proteobacterial classes and the soil properties in Caohai and Waihai sediments.**
(PDF)
