# Supplementary material for: Bacterial Communities in the Sediments of Dianchi Lake, a Partitioned Eutrophic Waterbody in China
Source: PLoS One. 2012 May 30;7(5):e37796. doi: 10.1371/journal.pone.0037796 (PMC3364273; doi:10.1371/journal.pone.0037796)

Figure S1 Map of sampling sites from Dianchi Lake, Yunnan Province, China.

The red line represents the dam that separates the Caohai (North part) and Waihai (South part). Sampling site of Caohai (▲):24°58'48.55"N, 102°38'31.92"E; Sampling site of Waihai (▼): 24°49'48.00"N, 102°42'47.00"E. The map was obtained from Google map and edited by the software Mapinfo 7.0.

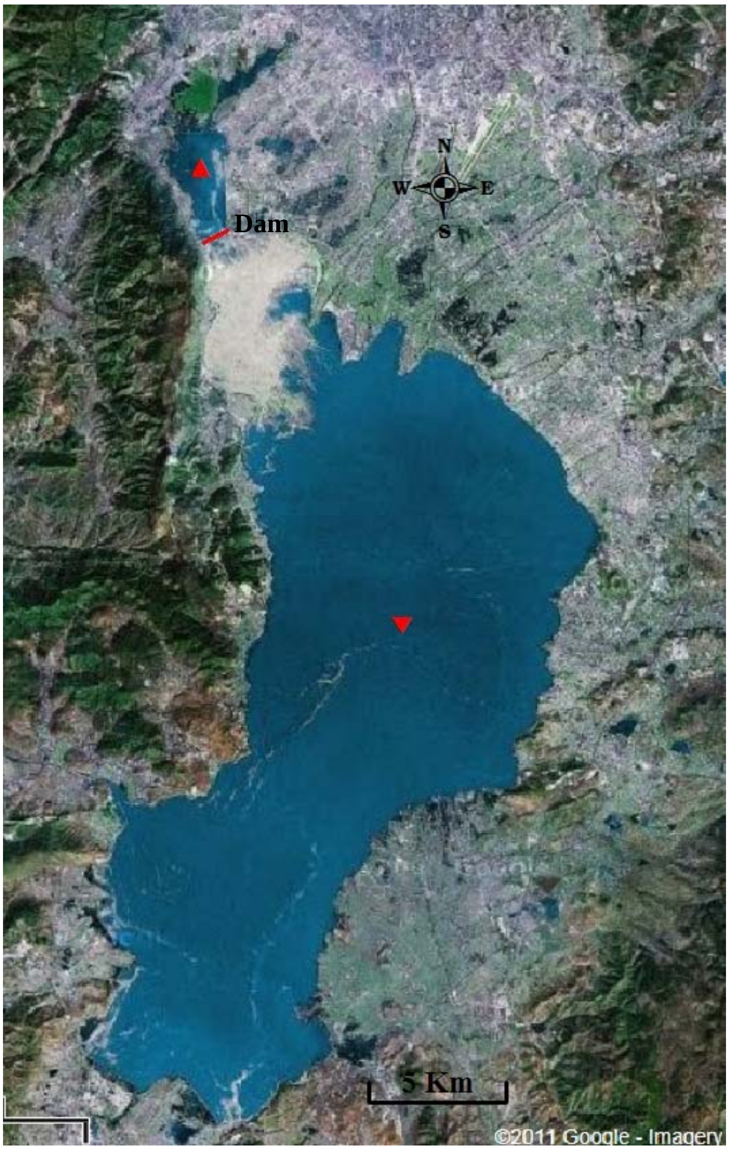

Supplement: Figure S1 — Map of sampling sites from Dianchi Lake, Yunnan Province, China. The red line represents the dam that separates the Caohai (North part) and Waihai (South part). Sampling site of Caohai (▴):24°58′48.55″N, 102°38′31.92″E; Sampling site of Waihai (▾): 24°49′48.00″N, 102°42′47.00″E. The map was obtained from Google map and edited by the software Mapinfo 7.0. (PDF) [file pone.0037796.s001.pdf]
