# Supplementary figures and images for: Bacterial Communities in the Sediments of Dianchi Lake, a Partitioned Eutrophic Waterbody in China
Source: PLoS One. 2012 May 30;7(5):e37796. doi: 10.1371/journal.pone.0037796 (PMC3364273; doi:10.1371/journal.pone.0037796)

Figure S2 Photo of freeze-dried sediments (Sampling in Dec. 2010)

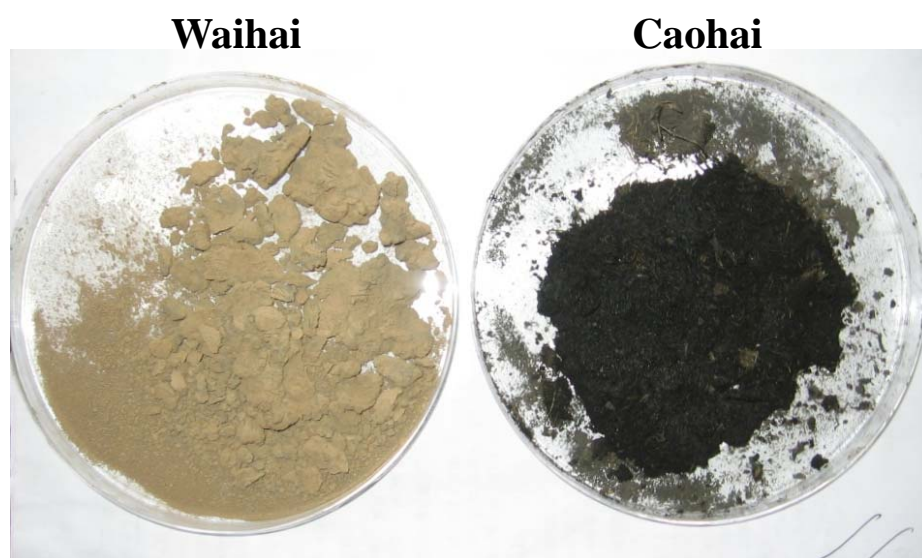

Supplement: Figure S2 — Photo of freeze-dried sediments (Sampling in Dec. 2010). (PDF) [file pone.0037796.s002.pdf]

Figure S3 Rarefaction curve of pyrosequencing libraries

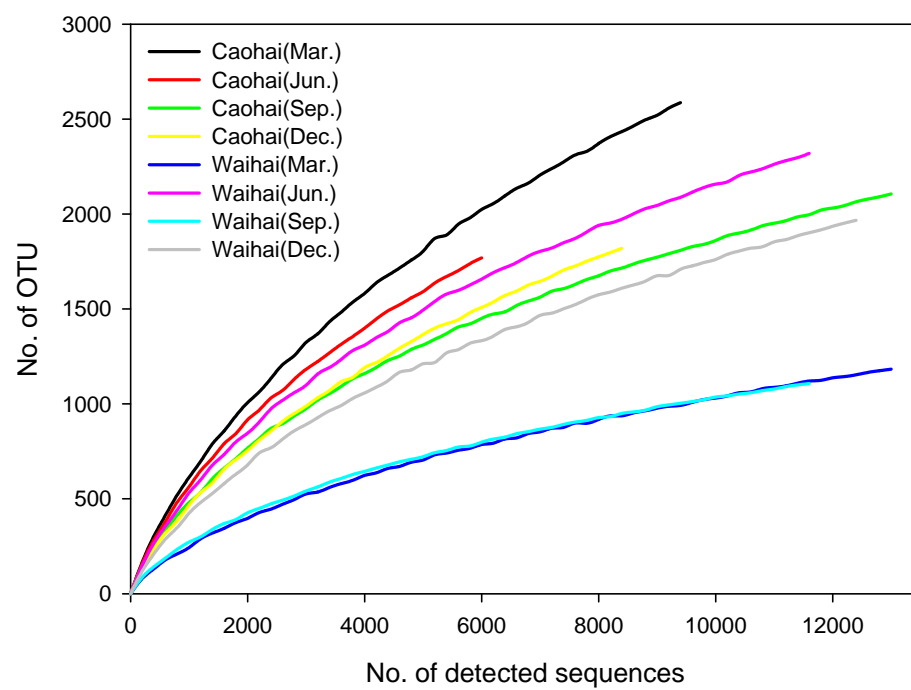

Supplement: Figure S3 — Rarefaction curve of pyrosequencing libraries. (PDF) [file pone.0037796.s003.pdf]

Figure S7 Rarefaction curve of *amoA* and *nosZ* clone libraries

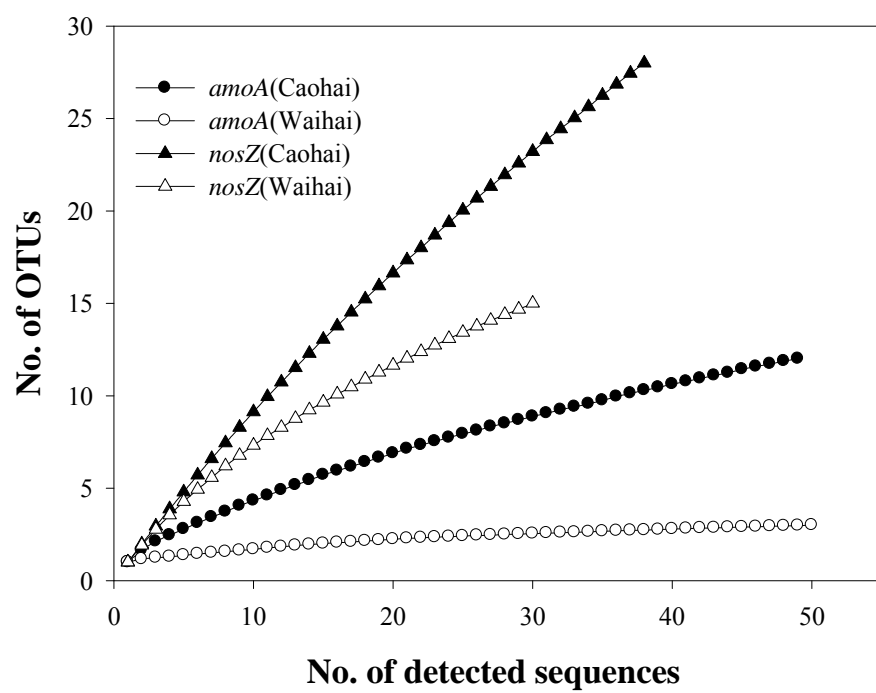

Supplement: Figure S7 — Rarefaction curve of amoA and nosZ clone libraries. (PDF) [file pone.0037796.s007.pdf]
