# Supplementary material for: Bacterial Communities in the Sediments of Dianchi Lake, a Partitioned Eutrophic Waterbody in China
Source: PLoS One. 2012 May 30;7(5):e37796. doi: 10.1371/journal.pone.0037796 (PMC3364273; doi:10.1371/journal.pone.0037796)

Figure S4 Hierarchical cluster analysis for barcoded pyrosequencing data based on Unweighted Pair Group Method using average linkage. The figure was plotted with FigTree software.

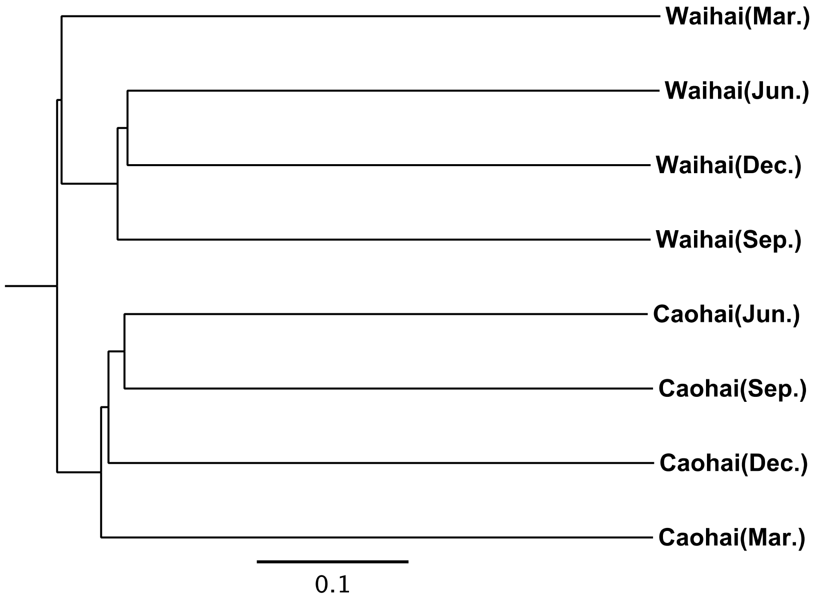

Supplement: Figure S4 — Hierarchical cluster analysis for barcoded pyrosequencing data based on Unweighted Pair Group Method using average linkage. The figure was plotted with FigTree software. (PDF) [file pone.0037796.s004.pdf]
