# Supplementary material for: Bacterial Communities in the Sediments of Dianchi Lake, a Partitioned Eutrophic Waterbody in China
Source: PLoS One. 2012 May 30;7(5):e37796. doi: 10.1371/journal.pone.0037796 (PMC3364273; doi:10.1371/journal.pone.0037796)

Figure S5 Relative abundances of the most abundant genera (top 10 of each sample) in Caohai and Waihai sediments. The heatmap was plotted with R program (http://www.r-project.org).

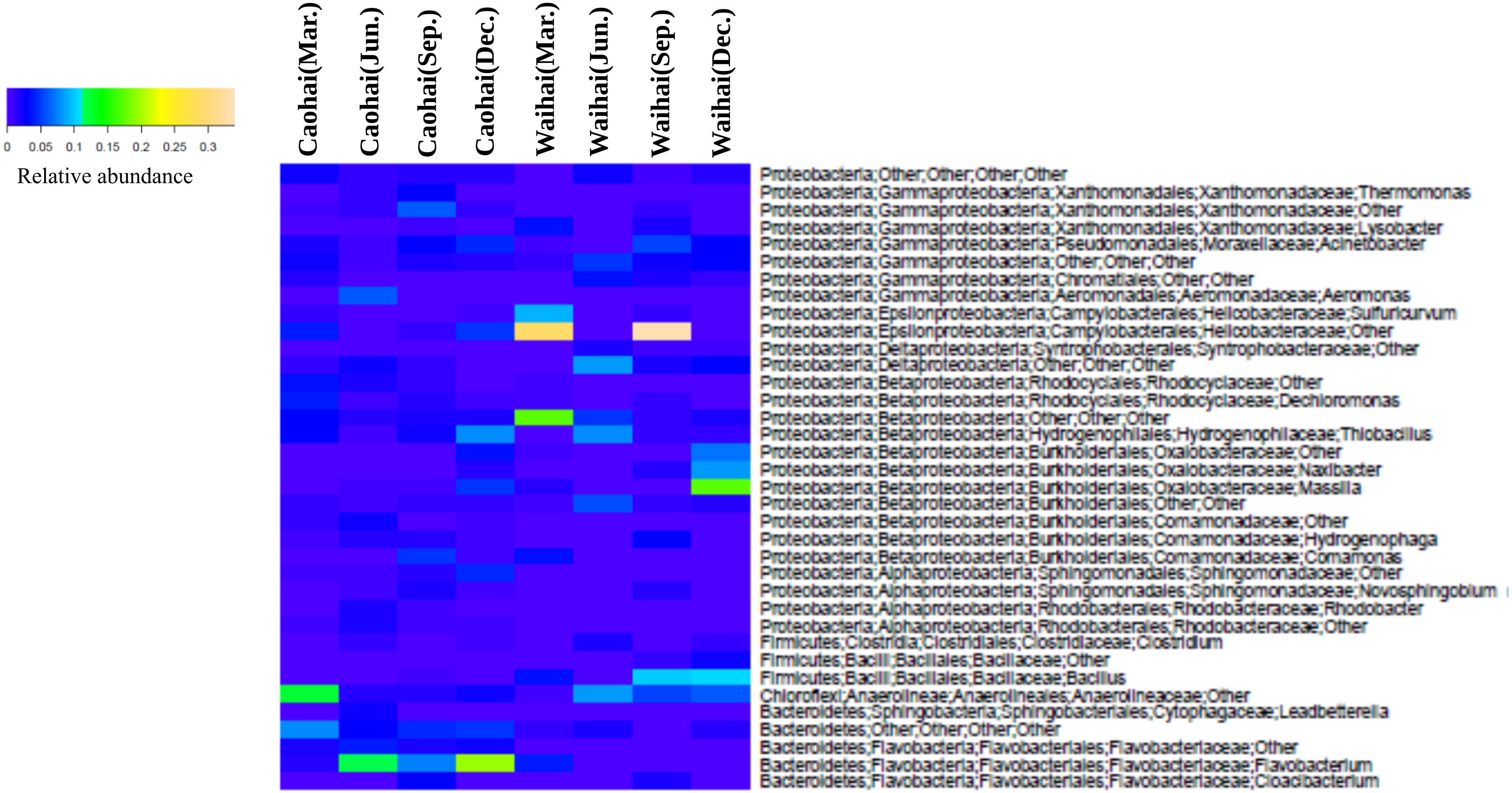

Supplement: Figure S5 — Relative abundances of the most abundant genera (top 10 of each sample) in Caohai and Waihai sediments. The heatmap was plotted with R program (http://www.r-project.org). (PDF) [file pone.0037796.s005.pdf]
