# Supplementary material for: Bacterial Communities in the Sediments of Dianchi Lake, a Partitioned Eutrophic Waterbody in China
Source: PLoS One. 2012 May 30;7(5):e37796. doi: 10.1371/journal.pone.0037796 (PMC3364273; doi:10.1371/journal.pone.0037796)
Supplement: Figure S6 — Dendrogram for hierarchical cluster analysis based on group-average linking of Bray-Curtis similarities calculated from the binary (0 or 1) data of the (a) amoA and (b) nosZ gene T-RFLP profiles. The symbol CH represents Caohai and WH represents Waihai. (PDF) [file pone.0037796.s006.pdf]

Figure S6 Dendrogram for hierarchical cluster analysis based on group-average linking of Bray-Curtis similarities calculated from the binary (0 or 1) data of the (a) *amoA* and (b) *nosZ* gene T-RFLP profiles. The symbol CH represents Caohai and WH represents Waihai.

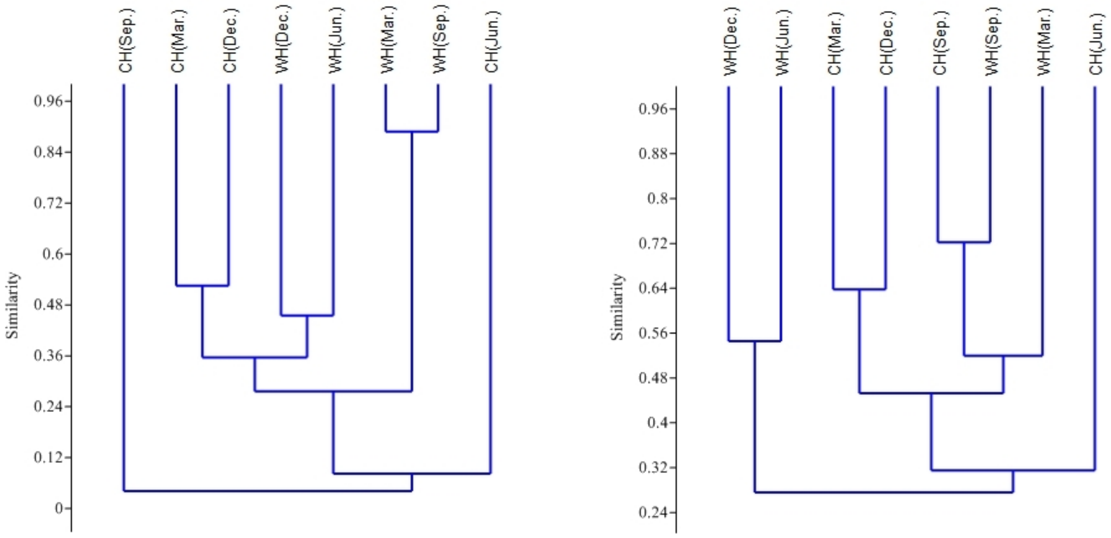

(a) *amoA* (b) *nosZ*
