# Supplementary material for: Bacterial Communities in the Sediments of Dianchi Lake, a Partitioned Eutrophic Waterbody in China
Source: PLoS One. 2012 May 30;7(5):e37796. doi: 10.1371/journal.pone.0037796 (PMC3364273; doi:10.1371/journal.pone.0037796)
Supplement: Figure S8 — Phylogenetic trees with the amoA representative sequences (OTUs) from Dianchi Lake sediments (bold letter) and lakeshore water [6] . The numbers on the branch nodes represent percentage of bootstrap resamplings based on 1000 replicates (only ≥50% are shown). The scale bar indicates the number of nucleotide substitutions per site. (PDF) [file pone.0037796.s008.pdf]

Figure S8 Phylogenetic trees with the *amoA* representative sequences (OTUs) from Dianchi Lake sediments (bold letter) and lakeshore water [6]. The numbers on the branch nodes represent percentage of bootstrap resamplings based on 1000 replicates (only ≥50% are shown). The scale bar indicates the number of nucleotide substitutions per site.

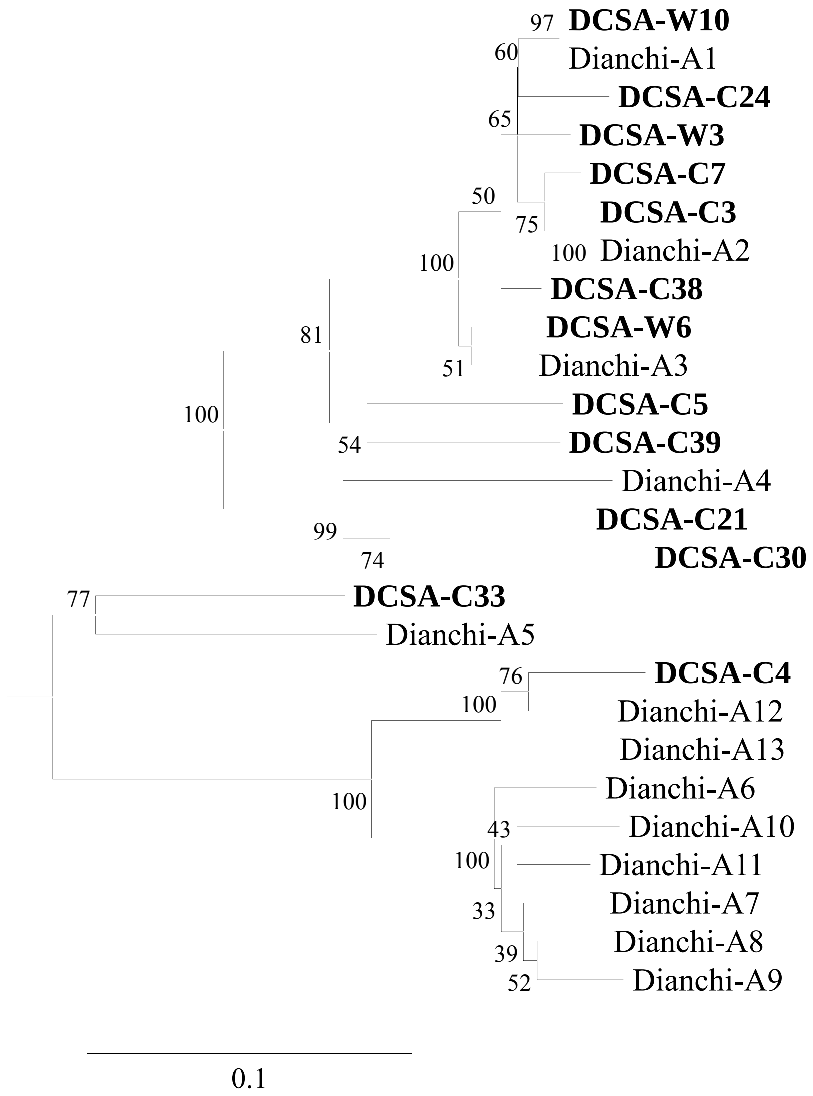

Reference

6. Wen DH, Bai YH, Shi Q, Li ZX, Sun QH, et al. (2011) Bacterial diversity in the polluted water of the Dianchi Lakeshore in China. Ann Microbiol DOI 10.1007/s13213-011-0311-9.
