# Supplementary material for: Bacterial Communities in the Sediments of Dianchi Lake, a Partitioned Eutrophic Waterbody in China
Source: PLoS One. 2012 May 30;7(5):e37796. doi: 10.1371/journal.pone.0037796 (PMC3364273; doi:10.1371/journal.pone.0037796)
Supplement: Table S1 — PCR primer pairs and thermal programs used in this study. (PDF) [file pone.0037796.s009.pdf]

Table S1 PCR primer pairs and thermal programs used in this study

Target gene	Sequence (5’-3’) of primer pairs	Thermal program	Reference
amoA(T-RFLP)	*amoA*-F: GGGGTTTCTACTGGTGGT<br>*amoA*-R: CCCCTCKGSAAAGCCTTCTTC	5 min at 94°C, 30-35 cycles (depend on the concentration of template) of 60 s at 94°C, 90 s at 60°C, and 90 s at 72°C, and a final cycle consisting of 90 s at 60°C and 10 min at 72°C	[1]
amoA (Clone library)		5 min at 94°C, 35 cycles of 60 s at 94°C, 90 s at 60°C, and 90 s at 72°C, and a final cycle consisting of 90 s at 60°C and 10 min at 72°C.	[1]
amoA (qPCR)		2 min at 95°C, followed by 40 cycles of 10 s at 94°C, 10 s at 55°C, and 30 s at 72°C.	[2]
nosZ (T-RFLP)	*nosZ*-F: CGYTGTTCMTCGACAGCCAG<br>*nosZ*-R: CATGTGCAGNGCRTGGCAGAA	3 min at 94°C, 1 cycle of 20 s at 94°C, 30 s at 65°C, 30 s at 72°C; 2 cycles of 20 s at 94°C, 30 s at 62°C, 35 s at 72°C; 3 cycles of 20 s at 94°C, 30 s at 59°C, 40 s at 72°C; 4 cycles of 20 s at 94°C, 30 s at 56°C, 45 s at 72°C; 5 cycles of 20 s at 94°C, 30 s at 53°C, 50 s at 72°C; 30 cycles of 20 s at 94°C , 45 s at 50°C, 1 min at 72°C; finally, 10 min at 72°C.	[3]
nosZ (Clone library)	*nosZ*-F: CGCRACGGCAASAAGGTSMSSGT<br>*nosZ*-R: CAKRTGCAKSGCRTGGCAGAA	5 min at 95°C, 35 cycles of 30 s at 95°C, 30 s at 60°C, and 30 s at 72°C, followed by 10 min at 72°C.	
nosZ (qPCR)		2 min at 95°C, 40 cycles of 10 s at 95°C, 40 s at 60°C.	[4]
16S rRNA (qPCR)	341F: CCTACGGGAGGCAGCAG<br>518R: ATTACCGCGGCTGCTGG	2 min at 95°C, 40 cycles of 10 s at 95°C, 40 s at 60°C.	[5]

References

1. Rotthauwe JH, Witzel KP, Liesack W (1997) The ammonia monooxygenase structural gene *amoA* as a functional marker: Molecular fine-scale analysis of natural ammonia-oxidizing populations. Appl Environ Microbiol 63: 4704-4712.
2. Chen XP, Zhu YG, Xia Y, Shen JP, He JZ (2008) Ammonia-oxidizing archaea: important players in paddy rhizosphere soil? Environ Microbiol 10: 1978-1987.
3. Rösch C, Mergel A, Bothe H (2002) Biodiversity of denitrifying and dinitrogen-fixing bacteria in an acid forest soil. Appl Environ Microbiol 68: 3818-3829.
4. Kong AYY, Hristova K, Scow KM, Six J (2010) Impacts of different N management regimes on nitrifier and denitrifier communities and N cycling in soil microenvironments. Soil Biol Biochem 42: 1523-1533.
5. He S, Gall DL, McMahon KD (2007) "*Candidatus accumulibacter*" population structure in enhanced biological phosphorus removal Sludges as revealed by polyphosphate kinase genes. Appl Environ Microbiol 73: 5865-5874.
