# Supplementary material for: Bacterial Communities in the Sediments of Dianchi Lake, a Partitioned Eutrophic Waterbody in China
Source: PLoS One. 2012 May 30;7(5):e37796. doi: 10.1371/journal.pone.0037796 (PMC3364273; doi:10.1371/journal.pone.0037796)
Supplement: Table S2 — Physico-chemical characteristics of the sediments of Dianchi Lake. (PDF) [file pone.0037796.s010.pdf]

Table S2 Physico-chemical characteristics of the sediments of Dianchi Lake

| Sample | Water content | Temperature (°C) | pH | TOC (mg/g) | $NH_3$-N (mg/g) | $NO_3^-$-N (mg/g) | $NO_2^-$-N (mg/g) |
|---|---|---|---|---|---|---|---|
| Caohai(Mar.) | 82.3% | 15.8 | 6.2 | 514.8 | 49.0 | 625.3 | 2.4 |
| Caohai(Jun.) | 91.5% | 22.9 | 6.2 | 1428.4 | 90.0 | 441.4 | 2.3 |
| Caohai(Sept.) | 91.4% | 21.8 | 6.4 | 9932.3 | 178.9 | 1113.2 | 7.7 |
| Caohai(Dec.) | 88.1% | 10.6 | 6.4 | 10915.0 | 389.2 | 1732.1 | 16.6 |
| Waihai(Mar.) | 63.8% | 15.5 | 6.2 | 350.0 | 58.6 | 1095.3 | 1.1 |
| Waihai (Jun.) | 79.1% | 22.3 | 6.4 | 450.5 | 118.7 | 568.2 | 0.6 |
| Waihai (Sep.) | 86.7% | 21.6 | 6.4 | 575.4 | 143.0 | 1291.4 | 10.9 |
| Waihai (Dec.) | 65.0% | 10.5 | 6.2 | 965.5 | 359.0 | 2795.2 | 12.2 |
