# Supplementary material for: Bacterial Communities in the Sediments of Dianchi Lake, a Partitioned Eutrophic Waterbody in China
Source: PLoS One. 2012 May 30;7(5):e37796. doi: 10.1371/journal.pone.0037796 (PMC3364273; doi:10.1371/journal.pone.0037796)
Supplement: Table S3 — Library coverage estimations and sequence diversity of amoA and nosZ . (PDF) [file pone.0037796.s011.pdf]

Table S3 Library coverage estimations and sequence diversity of *amoA* and *nosZ*

| Sample | No. of clones sequenced | No. of filtered sequences | No. of OTU (>98% identity) | % Coverage | Chao 1 value | Shannon index |
|---|---|---|---|---|---|---|
| Caohai(*amoA)* | 50 | 49 | 12 | 85.7% | 19(13, 49) | 1.53 |
| Caohai(*nosZ*) | 50 | 38 | 28 | 42.1% | 85(47, 207) | 3.21 |
| Waohai(*amoA)* | 50 | 50 | 3 | 98.0% | 3(3, 0) | 0.32 |
| Waohai(*nosZ*) | 50 | 30 | 15 | 70.0% | 24(17, 55) | 2.45 |

Library coverage was calculated as $C=1-n/N$, where $n$ is the number of OTUs without a replicate, and $N$ is the total number of sequences. The Shannon index = $-\sum p_i \ln p_i$ , where $p_i = n_i/N$, $n_i$ is the number of OTUs with $i$ individuals, and $N$ is the total number of individuals. The numbers in parentheses are lower and upper 95% confidence intervals for the Chao 1 estimators.
