# Supplementary material for: Bacterial Communities in the Sediments of Dianchi Lake, a Partitioned Eutrophic Waterbody in China
Source: PLoS One. 2012 May 30;7(5):e37796. doi: 10.1371/journal.pone.0037796 (PMC3364273; doi:10.1371/journal.pone.0037796)
Supplement: Table S4 — Correlations between richness (Chao 1 values), diversity (Shannon index), abundance of 16 S rRNA, amoA , nosZ (qPCR) and sediment properties. (PDF) [file pone.0037796.s012.pdf]

Table S4. Correlations between richness (Chao 1 values), diversity (Shannon index), abundance of 16S rRNA, *amoA*, *nosZ* (qPCR) and sediment properties.

	Richness	Diversity	Abundance		
			$Log_{10}{}^{16S\ rRNA}$	$Log_{10}{}^{amoA}$	$Log_{10}{}^{nosZ}$
pH	-0.218	-0.109	0.109	-0.218	0.109
TOC	0.119	0.167	**0.738***	0.333	0.619
NH_3-N	-0.357	-0.333	0.190	-0.214	0.024
NO_3^--N	-0.619	-0.619	-0.286	-0.548	-0.381
NO_2^--N	-0.286	-0.262	0.167	-0.143	0.119
Temperature	0.381	0.524	0.381	0.357	0.429

Significance at *α= 0.05 level
