# Supplementary material for: Bacterial Communities in the Sediments of Dianchi Lake, a Partitioned Eutrophic Waterbody in China
Source: PLoS One. 2012 May 30;7(5):e37796. doi: 10.1371/journal.pone.0037796 (PMC3364273; doi:10.1371/journal.pone.0037796)
Supplement: Table S5 — Correlations between the relative abundances of the most abundant bacterial phyla and proteobacterial classes and the soil properties in Caohai and Waihai sediments. (PDF) [file pone.0037796.s013.pdf]

Table S5 Correlations between the relative abundances of the most abundant bacterial phyla and proteobacterial classes and the soil properties in Caohai and Waihai sediments.

	Temperature	pH	TOC	$NH_3+NO_2^-$-N+NO_3^--N
Acidobacteria	0.286	0.000	-0.310	-0.310
Actinobacteria	-0.027	-0.375	**-0.791***	-0.518
Bacteroidetes	-0.048	-0.109	**0.714***	-0.095
Chloroflexi	0.071	-0.109	-0.262	-0.190
Firmicutes	-0.643	-0.109	-0.167	0.690
Planctomycetes	-0.245	-0.375	-0.355	-0.082
Alphaproteobacteria	0.429	0.218	**0.762***	-0.286
Betaproteobacteria	**-0.833***	-0.436	-0.048	0.452
Deltaproteobacteria	0.262	-0.218	-0.381	-0.262
Epsilonproteobacteria	-0.366	0.112	-0.220	0.195
Gammaproteobacteria	**0.738***	0.546	0.357	-0.238
Unclassified *Proteobacteria*	-0.024	0.165	0.036	-0.096
Unclassified bacteria	0.524	-0.109	-0.048	-0.405

Significance at *α= 0.05 level
